# Supplementary material for: Physiological and Putative Organic Cation Transporter Expression Response to Alizarin Dye Exposure in Aedes aegypti Mosquitoes
Source: Insects. 2025 Nov 25;16(12):1196. doi: 10.3390/insects16121196 (PMC12734359; doi:10.3390/insects16121196)
Supplement: Supplementary file 1 [file insects-16-01196-s001.zip › insects-3955488-supplementary.pdf]

Supplemental Materials

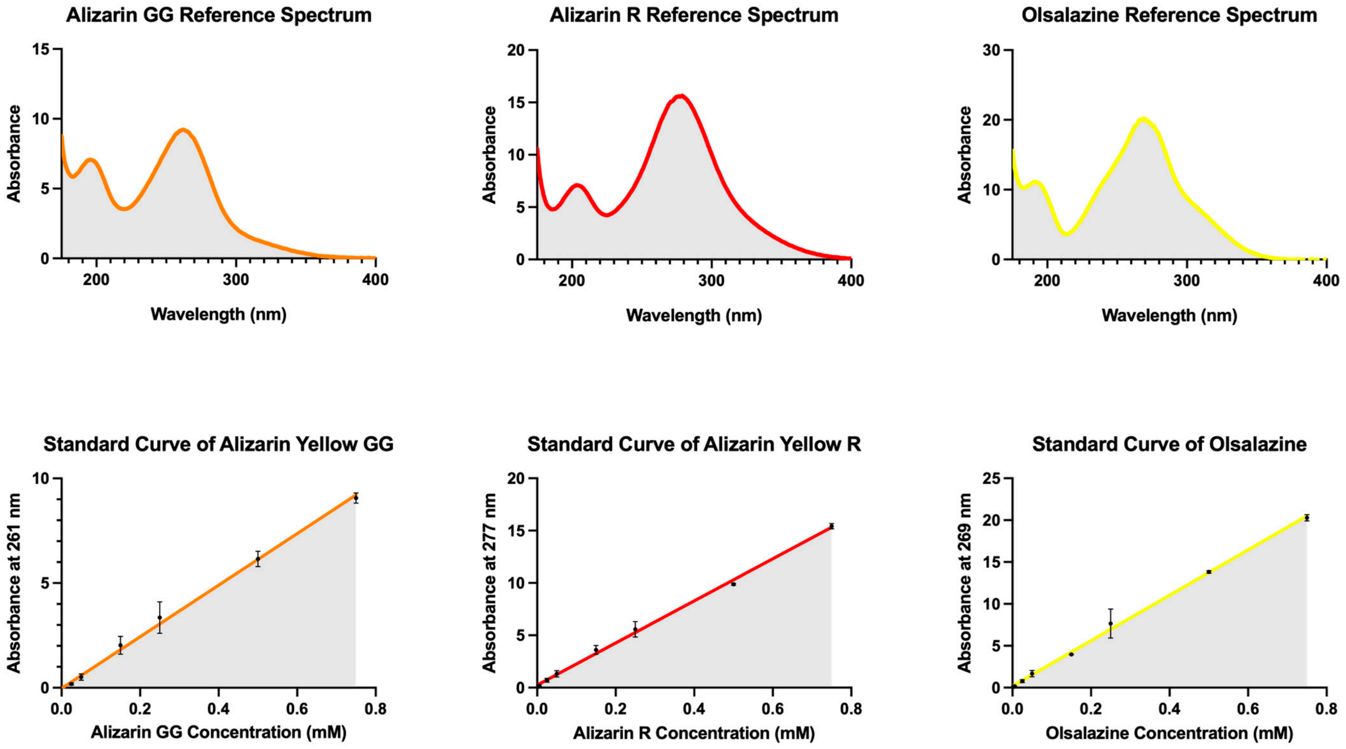

Figure S1. Absorbance spectra and standard curves of Alizarin Yellow GG, Alizarin Yellow R, and Olsalazine in PBS. Standard curves were generated by serial dilutions of each dye in PBS. Dilutions were performed in triplicate and absorbance was measured using a NanoDrop One Spectrophotometer.

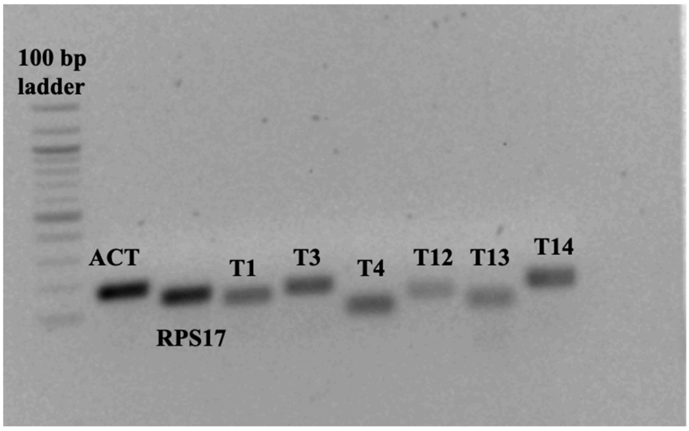

Figure S2. Gel electrophoresis of amplicons for qPCR. PCR products were separated on a 1% agarose gel. Ladder was Quick-Load® Purple 100 bp DNA Ladder (New England Biolabs, Ipswich, MA, USA). Each primer pair produced a single amplicon. ACT (*AAEL011197*), RPS17 (*AAEL004175*), T1 (*AAEL004451*), T3 (*AAEL012443*), T4 (*AAEL000902*), T12 (*AAEL024953*), T13 (*AAEL026837*), and T14 (*AAEL004479*).

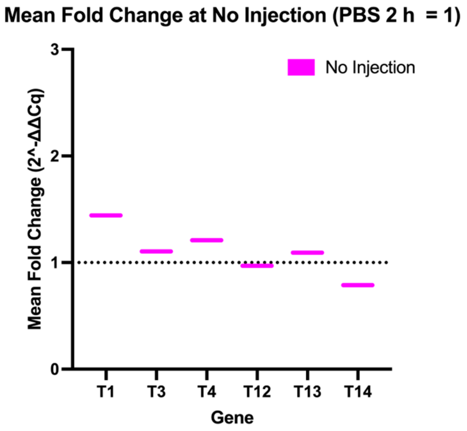

Figure S3. Preliminary qPCR of putative transporters: expression 2 hours after anesthetizing and wounding by a needle. Data is from one biological replicate, the genes were abbreviated as: T1 (*AAEL004451*), T3 (*AAEL012443*), T4 (*AAEL000902*), T12 (*AAEL024953*), T13 (*AAEL026837*), and T14 (*AAEL004479*).

```
Color Align Conservation results 70% for identity or similarity coloring
AAEL013489_TM LS------------------------------------------------------------------------------ 2
AAEL003192_TM Y---N--LIFNV---IGV-MFFAMTVVNVLLILTVP-EH-NCMVPGKEQ-Y-NV---------S-AIE-----RWRNLTL 52
AAEL003148_TM RF--N--LIFNM---GAI-IFASMSYMSIILALNKP-PH-NCHVPGMER-F-NI---------S-DAE-----MWKNLTL 53
AAEL024914_TM IL--F--LL-MI---PFA-FFVAFVYFSQIFITIVPEEH-WCWVPELEH-L-SV-------------E-----ERRALAI 50
AAEL013271_TM LL--L--WLICL---PAC-IPCGFCAFNQLFMTDVPEDY-WCKIPELQN-F-SV-------------E-----ERKLYGI 51
AAEL022769_TM LV--I--CVLLL---PAV-IPCAFHAYSQLFIAAIP-NH-WCRIPELEK-V-QP----------WAST-----LAKELSI 53
AAEL011275_TM LV--I--CVLLL---PAV-IPCAFHAYSQLFIAAIP-NH-WCRIPELEK-V-QP----------WAST-----LAKELSI 53
AAEL009206_TM RQ--Y--GLMVI----PI-ILNAFFTLSYVFTAGNL-NY-RCEVPGCDN-G-PN---------SVYHP-----EWLNNTV 53
CT2_TM        RVL-Y--FICAF----QN-ISCGIHYLASVFMGVTP-HH-VCRPPGNVSQV-VFHNHSNWSLEDT-GALLSSGQKDYVTV 68
AAEL000902_TM F-Q-F--VLHLL----AA-VTAGLHMLSLVTVAAVP-EH-RCFIEGVDS-A-SF---------NT-TSSMLDYNALGEYI 57
AAEL012443_TM KI--Y--VLLCL----PA-ISCAFHKLAGVFLLATP-DY-RCQLPFEADNV-TY---------QL-PP-----ELMSMAY 53
AAEL024953_TM L---F--ILCLT----PN-ILNGFHVSSYVFLGQMPENY-YCVVPSLVQ-R-G-----------WTHD-----EIRNISI 51
AAEL026837_TM YW--T--FLLCL----FQ-IPTTFHIFCLVFQAANR-DF-WCARPAHLRSI-PL-------------E-----LWRNLTH 50
AAEL004479_TM FVM-T--FLLSL----FQ-VPNTFHIYSPTFQAAEK-SH-WCRRPSHLNDI-SV-------------D-----LWRNVTQ 51
OCTN1_TM      LI--F--FL-LS---ASI-IPNGFNGMSVVFLAGTP-EH-RCRVPDAAN-L-SS-------------------AWRNNSV 48
OCTN2_TM      LI--F--FL-LS---ASI-IPNGFTGLSSVFLIATP-EH-RCRVPDAAN-L-SS-------------------AWRNHTV 48
OCT1_TM       AF-----LILCL----LS-AAFAPICVGIVFLGFTP-DH-HCQSPGVAE-L-SQ--RCG-----WSPA-----EELNYTV 54
OCT2_TM       QM--F--FLLAL----LS-ATFAPIYVGIVFLGFTP-DH-RCRSPGVAE-L-SL--RCG-----WSPA-----EELNYTV 55
OCT3_TM       VF-----LLLCL----TG-VTFAFLFVGVVFLGTQP-DHYWCRGPSAAA-L-AE--RCG-----WSPE-----EEWNRTA 55
AAEL004451_TM LGWWH--ILVCAVVFPLK-FPVAWHQMGIIFLGAAM-NY-TCSSNSSL-------------------------------- 43
OAT10_TM      IQL-L--ILLCV----LN-FLSPFYFFAHVFMVLDE-PH-HCAVAWVKN-H-TF---------NLSAA-----EQLVLSV 54
OAT2_TM       RN--V--ALLAL----PR-VLLPLHFLLPIFLAAVP-AH-RCALPGAPANF-S------------HQD-----VWLEAHL 51
OAT6_TM       NH--T--ALLLL----PC-GLLACHNFLQNFTAAVP-PH-HCRGPANHT-EAS----------TNDSG-----AWLRATI 53
OAT3_TM       FLH-V--AILGL----PI-LNMANHNLLQIFTAATP-VH-HCRPPHNAS-T----------------G-----PW---VL 45
OAT1_TM       QIQ-V--TLVVL----PL-LLMASHNTLQNFTAAIP-TH-HCRPPADAN-L-S-------------KN-----GGLEVWL 50
OAT5_TM       MLH-L---VFIL----PSLMLLIPHILLENFAAAIP-GH-RCWVHMLDN-N-TG---SGNETGILSED-----ALLRISI 60
OAT7_TM       FQI-LQTVFLSI----FA-VATYLHFMLENFTAFIP-GH-RCWVHILDN-D-TV---SDNDTGALSQD-----ALLRISI 62
URAT1_TM      VLQ-T---MALM----VSIMWLCTQSMLENFSAAVP-SH-RCWAPLLDN-S-TA---QASILGSLSPE-----ALLAISI 60
OAT4_TM       LQV-L---TFIL----PC-LMIPSQMLLENFSAAIP-GH-RCWTHMLDN-G-SA---V---STNMTPK-----ALLTISI 56
TM Domains           i[-TM1-----------------]o
```

Figure S4. Amino acid sequence alignment of the putative organic cation transporters and putative organic cation transporter novel from *Aedes aegypti* with human SLC22A. Human SLC22A proteins are in Bold. Similarity and identity threshold was set at 70% using the Color Align Conservation feature of the Sequence Manipulation Suite (Stothard, P. The Sequence Manipulation Suite: JavaScript Programs for Analyzing and Formatting Protein and DNA Sequences. *Biotechniques* **2000**, 28, 1102–1104). The top line indicates the strongly conserved regions among SLC22A peptides "^" and positions of class specific amino acids "*". The bottom line indicated the predicted transmembrane domains by CCTOP where "i" signals intracellular loops and "o" signals extracellular loops.

```
AAEL013489_TM ------------------------------------------------------------------------LCV--GLC 8
AAEL003192_TM PRE-PNS-----R-GELS-YSSCAMYNIS-----KY-PI----GTSP------NE--------WKFEANETISCI--Y-G 97
AAEL003148_TM PRE-EDN-----K-GVLS-YSKCQMYNVT-----EA-HL----QVHY------SE--------WNFQDSDIINCA--Y-G 98
AAEL024914_TM PVQ-YEHGTEFDD-GIAS-YSKCSMYAVN-----YT-EVL----ANN------IR--------KADPSWPIQSCK--N-G 100
AAEL013271_TM PTV-ER------D-GAEE-FSKCTRYAVD-----WK-ALL----AEN--DPST-I--------APNSSWPVELCR--D-G 98
AAEL022769_TM PLE-YK------D-GRSK-LAMCSMYSRN-----YS-QIANVLNAEN---S-TAV--------FKDPFDNITSCV--Y-G 103
AAEL011275_TM PLE-YK------D-GRSK-LAMCSMYSRN-----YS-QIANVLNAEN---S-TAV--------FKDPFDNITSCV--Y-G 103
AAEL009206_TM PFR------------HGI-PAQCERFQPI-----TN-ASL----DNL---L---------CDADDFDQKNIIEC---G-S 94
CT2_TM        QLQ---------NGEIWE-LSRCSRNKRE-----NT-SSL----G-----------YE------YTGSKKEFPCV--D-G 108
AAEL000902_TM PLN-----------KDGE-LESCWMFD---------------------------------------G-SNRTTC---T-S 81
AAEL012443_TM PMD-----------SFSKKYSTCTYLDAN-----F--TDE----YMN---G-G------------TPAKEVASC---D-H 91
AAEL024953_TM IA------------GSAK-NGTCSLLNWN-----YH-ALE----G--------MNYQEAQEYLKDKPLPGEVSCLRTD-G 99
AAEL026837_TM P-----------N------GGQCSHLNAP-----YE-QLS----LQNFTDY-FGS--------VNVSTLEQVSC---N-G 90
AAEL004479_TM Q------------------RENCRILNYD-----WN-SV------GSEQTL-RNL--------SIPGNLSHVG ---S-S 88
OCTN1_TM      PLR-LR------D-GREV-PHSCSRYRLA-----TI-ANFSALGLEP---G-R-D--------VDLGQLEQESCL--D-G 97
OCTN2_TM      PLR-LR------D-GREV-PHSCRRYRLA-----TI-ANFSALGLEP---G-R-D--------VDLGQLEQESCP--D-G 97
OCT1_TM       PGL-GP------A-GEAF-LGQCRRYEVD-----WN-QS-ALSCVDP--LA-SLA--------TNRSHLPLGPCQ--D-G 104
OCT2_TM       PGP-GP------A-GEAS-PRQCRRYEVD-----WN-QS-TFDCVDP--LA-SLD--------TNRSRLPLGPCR--D-G 105
OCT3_TM       PASRGP------E-PPER-RGRCQRYLLEAANDSAS-AT-SALSCAD--PL-AAF--------PN-RSAPLVPCR--G-G 110
AAEL004451_TM --------------------DRC-----------------------------------------------SSEC---T-S 52
OAT10_TM      PLD-----------TAGH-PEPCLMFRPP-----PANASL----QDI---LS-------------HRFNETQPCD--M-G 94
OAT2_TM       PRE-----------PDGT-LSSCLRFAYP-----QA-L-------PN---T-T-LGEERQSRGELEDEPATVPCS--Q-G 98
OAT6_TM       PLD-----------QLGA-PEPCRRFTKP-----QW-ALL----SPN---S-S-I--------P---GAATEGCK--D-G 92
OAT3_TM       PMG-----------PNGK-PERCLRFVHP-----P-----------N---A-S-L--------PNDTQRAMEPCL--D-G 81
OAT1_TM       PRD-----------RQGQ-PESCLRFTSP-----QW-GLP----FLN---G-T-E--------AN-GTGATEPCT--D-G 91
OAT5_TM       PLD-----------SNLR-PEKCRRFVHP-----QW-QLL----HLN---G-T-I--------HSTSEADTEPCV--D-G 102
OAT7_TM       PLD-----------SNMR-PEKCRRFVHP-----QW-QLL----HLN---G-T-F--------PNTSDADMEPCV--D-G 104
URAT1_TM      PPG-----------PNQR-PHQCRRFRQP-----QW-QLL----DPN---A-T-A--------TSWSEADTEPCV--D-G 102
OAT4_TM       PPG-----------PNQG-PHQCRRFRQP-----QW-QLL----DPN---A-T-A--------TSWSEADTEPCV--D-G 98
TM Domains
```

Figure S4 continued. Amino acid sequence alignment of the putative organic cation transporters and putative organic cation transporter novel from *Aedes aegypti* with human SLC22A in Bold. Similarity and identity threshold was set at 70%. The top line indicates the strongly conserved regions among SLC22A peptides "^" and positions of class specific amino acids "*". The bottom line indicated the predicted transmembrane domains by CCTOP where "i" signals intracellular loops and "o" signals extracellular loops.

```
              *                            *   *                        ^^^*^^^^^^
AAEL013489_TM WM-ADSMEMTILSV-LGPALHC--DWGITR----YQQALVTTVVFLGMMLSSTFWGHLSDRYGRKPALTLCGVLLILYGL 80
AAEL003192_TM YE-YDRQ------Y-FDLIPIT--EENWVCDKE-LHATNIFAFIRFAEVSGTFVLGQLGDRIGRLPVYLLSIGAATIGRT 166
AAEL003148_TM YD-YDRT------Y-YDRTPIT--EHDWICDKG-FRETNIFVYNRFGELIGTFIFGQLGDTLGRRPVFFFSVLIITAGRL 167
AAEL024914_TM WE-YNFT------DVPYRTAAT--DFEWVCENA-YLSTLSQSIFFVGAIVGGLLFGWIADRYGRIPALAGCNVIGFVAGV 170
AAEL013271_TM WD-YNSS------I-VMSSIVI--DYNLVCEYD-IYPTLGLVALNTGGPIGVYLFGMLNDRAGRRMAYFCCLATLLFGSF 167
AAEL022769_TM WK-YDTS------V-FESTVVT--EWNLVCEKE-FYPTAALIVFGIGGLVGNLVFGYMQDYWGRKNSFFVYLFIEVAACA 172
AAEL011275_TM WK-YDTS------V-FESTVVT--EWNLVCEKE-FYPTAALIVFGIGGLVGNLVFGYMQDYWGRKNSFFVYLFIEVAACA 172
AAEL009206_TM FI-FEDD---------EVTIVN--DFNITCPNNDWKLTLVGTINNVGQFVALPIAGYISDRFGRRLVLLLSVAGSAIFGV 162
CT2_TM        YI-YDQN------T-WKSTAVT--QWNLVCDRK-WLAMLIQPLFMFGVLLGSVTFGYFSDRLGRRVVLWATSSSMFLFGI 177
AAEL000902_TM YV-YNTT------Y-YKSSRTM--EWDLVCDRK-WMGALAQTVYMLGVFTGAVWLGGLADKIGRKKVFCWSGLLQLILGV 150
AAEL012443_TM WI-YDRS------K-FESSTVT--EWDLVCDRN-WLRASADSLFMVGVMLGSIIFGWLSDKYGRKPIFFASLVTQVIFGV 160
AAEL024953_TM LEMYYHQ------S-KGASIVP--EWDLICERT-ALRSTVQVALSLGKFFGASTFGILSDKYGRKSSFTIAALLYVVSSL 169
AAEL026837_TM YE-FDSR------I-FGQTVIT--EFGLVCDRA-QMMSIVEMCFLAGAAMGAVGSGWISDQFGRRHTLMCFALIQAVTGF 159
AAEL004479_TM WE-FDLN-----DN-LGNTWAS--EWSLVCDKE-YLKDVAEMFFLVGVATGGITSGVLSDKFGRKKMLFISAVLQAIFGL 158
OCTN1_TM      WE-FSQD------V-YLSTVVT--EWNLVCEDN-WKVPLTTSLFFVGVLLGSFVSGQLSDRFGRKNVLFATMAVQTGFSF 166
OCTN2_TM      WE-FSQD------V-YLSTIVT--EWNLVCEDD-WKAPLTISLFFVGVLLGSFISGQLSDRFGRKNVLFVTMGMQTGFSF 166
OCT1_TM       WV-YDTP---------GSSIVT--EFNLVCADS-WKLDLFQSCLNAGFLFGSLGVGYFADRFGRKLCLLGTVLVNAVSGV 171
OCT2_TM       WV-YETP---------GSSIVT--EFNLVCANS-WMLDLFQSSVNVGFFIGSMSIGYIADRFGRKLCLLTTVLINAAAGV 172
OCT3_TM       WR-YAQA---------HSTIVS--EFDLVCVNA-WMLDLTQAILNLGFLTGAFTLGYAADRYGRIVIYLLSCLGVGVTGV 177
AAEL004451_TM WN-YDTS------V-FSSTIVS--EWNLVCEKA-NLVNLSQTIFMFGILVGGVLFGSLADKFGRRPPLVIAVIIQLISGV 121
OAT10_TM      WE-YPEN------R-L-PSLKN--EFNLVCDRK-HLKDTTQSVFMAGLLVGTLMFGPLCDRIGRKATILAQLLLFTLIGL 162
OAT2_TM       WE-YDHS------E-FSSTIATESQWDLVCEQK-GLNRAASTFFFAGVLVGAVAFGYLSDRFGRRRLLLVAYVSTLVLGL 169
OAT6_TM       WV-YNRS------V-FPSTIVM--EWDLVCEAR-TLRDLAQSVYMAGVLVGAAVFGSLADRLGCKGPLVWSYLQLAASGA 161
OAT3_TM       WV-YNST---------KDSIVT--EWDLVCNSN-KLKEMAQSIFMAGILIGGLVLGDLSDRFGRRPILTCSYLLLAASGS 148
OAT1_TM       WI-YDNS------T-FPSTIVT--EWDLVCSHR-ALRQLAQSLYMVGVLLGAMVFGYLADRLGRRKVLILNYLQTAVSGT 160
OAT5_TM       WV-YDQS------Y-FPSTIVT--KWDLVCDYQ-SLKSVVQFLLLTGMLVGGIIGGHVSDRFGRRFILRWCLLQLAITDT 171
OAT7_TM       WV-YDRI------S-FSSTIVT--EWDLVCDSQ-SLTSVAKFVFMAGMMVGGILGGHLSDRFGRRFVLRWCYLQVAIVGT 173
URAT1_TM      WV-YDRS------I-FTSTIVA--KWNLVCDSH-ALKPMAQSIYLAGILVGAAACGPASDRFGRRLVLTWSYLQMAVMGT 171
OAT4_TM       WV-YDRS------V-FTSTIVA--KWDLVCSSQ-GLKPLSQSIFMSGILVGSFIWGLLSYRFGRKPMLSWCCLQLAVAGT 167
TM Domains                                      o[-TM2--------------]i      i[-TM3---------
```

Figure S4 continued. Amino acid sequence alignment of the putative organic cation transporters and putative organic cation transporter novel from *Aedes aegypti* with human SLC22A in Bold. Similarity and identity threshold was set at 70%. The top line indicates the strongly conserved regions among SLC22A peptides "^" and positions of class specific amino acids "*". The bottom line indicated the predicted transmembrane domains by CCTOP where "i" signals intracellular loops and "o" signals extracellular loops.

```
                                                  ^^^^^^^^
AAEL013489_TM LSSVAP-SFGWLLLLRGLVGF-AIG-CVPQSVTLYAEFLPTKQRAKCVVL-LDCFWALGACFEVALALAVGPNLGWRWLL 156
AAEL003192_TM LAVLTASQYWLFAALAFMGSF-ASNTAFQSPLIVAMEISKDENRAALSLW-QLFGWTVGVCVAPLILWWT---RNWIWFM 241
AAEL003148_TM ISMFTASIYVVFCMAAVLGSL-TSNSIFQAPLIIAMEVSKSERRGYISMM-QCIGWTTGLCILPMVFWAT---RDWFWAL 242
AAEL024914_TM VTAFVG-NFWQFALCRFLVGF-AFDNCFTMMYILVLEYVGPKWRTFVANMSIALFFTAASCALPWIAYYL---ANWKIFA 245
AAEL013271_TM ITAASS-SFWMWAFSRVIVGL-TIPAVYQIPFIIALELVGPNYRSFVTVM-TCTFYTFGLMMLAGVTYLI---RDWVELT 241
AAEL022769_TM SGVFTW-NFELWLLTRFIVGL-TVPAILSSPYVLAIELVEPRKRDFCTIV-SNIAYSIGLIMLAGVVYLF---RDWRSLS 246
AAEL011275_TM SGVFTW-NFELWLLTRFIVGL-TVPAILSSPYVLAIELVEPRKRDFCTIV-SNIAYSIGLIMLAGVVYLF---RDWRSLS 246
AAEL009206_TM IRSFAA-SYEMFIALEFLDPL-IGSTMYTTAFVLALELVGPKMRVTGNNI-ISCAFSFAEAGLGLLAMLL---RNWRYLL 236
CT2_TM        AAAFAV-DYYTFMAARFFLAM-VASGYLVVGFVYVMEFIGMKSRTWASVH-LHSFFAVGTLLVALTGYLV---RTWWLYQ 251
AAEL000902_TM AVAFTP-EYYSFLVIRYLYGIFGSAGSYITGFVLTMELVGPSKRTPCGIS-FQAAFAGGIILVAAWGAVI---HDRMLLQ 225
AAEL012443_TM LAGIAP-EFFTYTIARILVGA-TTSGVFLVAYVIAMEMVGPKDRLYAGVV-CMMFFSVGYMLTAGFAYFI---HDWRTLQ 234
AAEL024953_TM LTTFSP-VYILLLLGRIGLGA-SASGVFYPAFALLTENIGTRHRSWMSIA-FNFSYPIGMLILALAAYYI---HPWRDLS 243
AAEL026837_TM LLGFVT-SLELYMALRVIIGF-ASMSVAVVSFVLVVELVSGKYRTIIGIL-NILPVAISYVLAAAIAFFT---RDWRLMQ 233
AAEL004479_TM ALYFVD-SLEFLLILRALLGI-VSVSVTYAGLILAIEYVDGKWRTIAGMY-NLFPLPVSYIMISGLAYLT---QDFRSLQ 232
OCTN1_TM      LQIFSI-SWEMFTVLFVIVGM-GQISNYVVAFILGTEILGKSVRIIFSTLGVCTFFAVGYMLLPLFAYFI---RDWRMLL 241
OCTN2_TM      LQIFSK-NFEMFVVLFVLVGM-GQISNYVAAFVLGTEILGKSVRIIFSTLGVCIFYAFGYMVLPLFAYFI---RDWRMLL 241
OCT1_TM       LMAFSP-NYMSMLLFRLLQGL-VSKGNWMAGYTLITEFVGSGSRRTVAIM-YQMAFTVGLVALTGLAYAL---PHWRWLQ 245
OCT2_TM       LMAISP-TYTWMLIFRLIQGL-VSKAGWLIGYILITEFVGRRYRRTVGIF-YQVAYTVGLLVLAGVAYAL---PHWRWLQ 246
OCT3_TM       VVAFAP-NFPVFVIFRFLQGV-FGKGTWMTCYVIVTEIVGSKQRRIVGIV-IQMFFTLGIIILPGIAYFI---PNWQGIQ 251
AAEL004451_TM ATAYIP-WFWGFVVLRFITAV-ATGGTMVTSFVLVMEIIGPKWRELFSVL-YQIPFNLGHLTLAGFAYFL---RDWHHLQ 195
OAT10_TM      ATAFVP-SFELYMALRFAVAT-AVAGLSFSNVTLLTEWVGPSWRTQAVVL-AQCNFSLGQMVLAGLAYGF---RNWRLLQ 236
OAT2_TM       ASAASV-SYVMFAITRTLTGS-ALAGFTIIVMPLELEWLDVEHRTVAGVL-SSTFWTGGVMLLALVGYLI---RDWRWLL 243
OAT6_TM       ATAYFS-SFSAYCVFRFLMGM-TFSGIILNSVSLVVEWMPTRGRTVAGIL-LGYSFTLGQLILAGVAYLI---RPWRCLQ 235
OAT3_TM       GAAFSP-TFPIYMVFRFLCGF-GISGITLSTVILNVEWVPTRMRAIMSTA-LGYCYTFGQFILPGLAYAI---PQWRWLQ 222
OAT1_TM       CAAFAP-NFPIYCAFRLLSGM-ALAGISLNCMTLNVEWMPIHTRACVGTL-IGYVYSLGQFLLAGVAYAV---PHWRHLQ 234
OAT5_TM       CAAFAP-TFPVYCVLRFLAGF-SSMIIISNNSLPITEWIRPNSKALVVIL-SSGALSIGQIILGGLAYVF---RDWQTLH 245
OAT7_TM       CAALAP-TFLIYCSLRFLSGI-AAMSLITNTIMLIAEWATHRFQAMGITL-GMCPSGIAFMTLAGLAFAI---RDWHILQ 247
URAT1_TM      AAAFAP-AFPVYCLFRFLLAF-AVAGVMMNTGTLLMEWTAARARPLVMTL-NSLGFSFGHGLTAAVAYGV---RDWTLLQ 245
OAT4_TM       STIFAP-TFVIYCGLRFVAAF-GMAGIFLSSLTLMVEWTTTSRRAVTMTV-VGCAFSAGQAALGGLAFAL---RDWRTLQ 241
TM Domains    TM3-]o    o[-TM4---------------]i             i[-TM5-------------]o         o[-
```

Figure S4 continued. Amino acid sequence alignment of the putative organic cation transporters and putative organic cation transporter novel from *Aedes aegypti* with human SLC22A in Bold. Similarity and identity threshold was set at 70%. The top line indicates the strongly conserved regions among SLC22A peptides "^" and positions of class specific amino acids "*". The bottom line indicated the predicted transmembrane domains by CCTOP where "i" signals intracellular loops and "o" signals extracellular loops.

```
                               ^^^^^^^^
AAEL013489_TM GLSAAPLFAFAVITPWLPESARYHVASGQSDKALTTLEQIAKDNK--RP-MLLGR---LV--------V-EG-----P--  214
AAEL003192_TM LMTSVPCLLFYCMPQYNIESPRWLASQGRYQDCIQQLKKIAKVNK--SK-FHLTV---EEL----KEKIPQK-----E--  304
AAEL003148_TM MFTTIPIFLFIFIPQYLIESPRWLATRGHYKRSMGELRKIANVNG--IRQLPFDE---RSL----EKMLSHR-----K--  306
AAEL024914_TM IVTSAPLALAILTPWLVPESARWLVSQGKVDKAINILKKFEKINR--KT-VDAKV---YQDFSESCVRLQEE----EA--  313
AAEL013271_TM LYTSVPFLLYFLYLFIMPESPRWLLMKGKLEEALQVLEKMAKVNG--KQ-FPVAF---KNKLQK-RVQAEKD-RTVKR--  311
AAEL022769_TM LAVSLPLLLLFAFYCFIPESPRWLVARNRFKDAAQVMTIMAKLNR--KS-IPTNY---EHTLKC-KLSVPTD-VT-DK--  315
AAEL011275_TM LAVSLPLLLLFAFYCFIPESPRWLVARNRFKDAAQVMTIMAKLNR--KS-IPTNY---EHTLKC-KLSVPTD-VT-DK--  315
AAEL009206_TM RALYIPGIVSLPFLWMTTESVRWLLSKGQREKAFDVLKRAAKMNG--KT-LSPAA---IDSFCP----VSDNESR-SE--  303
CT2_TM        MILSTVTVPFILCCWVLPETPFWLLSEGRYEEAQKIVDIMAKWNR--AS-SC-KLSELLSLDLQ--GPVSNS----PT--  319
AAEL000902_TM VIYGLHGLLLIAHWWVMDESPRWLWMQNRKREAIDIIAKAVRMNG--RG-LSVDKEYYLSKDK---SNFSAE----AT--  293
AAEL012443_TM IALTLPGILFMTYWWLIPESSRWLISNNRPSEAITLIKKVAKSNK--VT-VPEDV---LDKLVE-EDKAALE-S--DK--  302
AAEL024953_TM LALTIPSFLLVIHLYFLVESPRWLLSKGHERRAYRMVFGRKAPEELCDS-AEKDQ---LSPEEV-AAAVAKD-V--PPPK 315
AAEL026837_TM FVITTPGACLLLMWYWCPESPRWLLAKGRIEELKAIIDQAARVNG--FK-LPSGY---EKTL-----QVPET----ET--  296
AAEL004479_TM LCIGLPGVFLCFLWFVLPESPRWLLCKGRIAEVKEIVRKAAAFNN--RP-LPDNL---DKLL-----KPP-T----DE--  294
OCTN1_TM      LALTVPGVLCVPLWWFIPESPRWLISQRRFREAEDIIQKAAKMNN--IA-VPAVI---FDS-----V---EE-LN-PL--  303
OCTN2_TM      VALTMPGVLCVALWWFIPESPRWLISQGRFEEAEVIIRKAAKANG--IV-VPSTI---FDPSE---L---QD-LS-SK--  305
OCT1_TM       LAVSLPTFLFLLYYWCVPESPRWLLSQKRNTEAIKIMDHIAQKNG--KL-PPADL---KML------SLEED-V---T--  307
OCT2_TM       FTVSLPNFFFLLYYWCIPESPRWLISQNKNAEAMRIIKHIAKKNG--KS-LPASL---QRL------RLEEE-T---G--  308
OCT3_TM       LAITLPSFLFLLYYWVVPESPRWLITRKKGDKALQILRRIAKCNG--KY-LSSNY---SEI------TVTDE--------  311
AAEL004451_TM FGLSIFSLLLVSYYWLVPESPRYLFTSGNTEKAVQLLETAAKRNN--LP-TEN-I---RSDI-E-QY-AKTK-T--HG--  260
OAT10_TM      ITGTAPGLLLFFYFWALPESARWLLTRGRMDEAIQLIQKAASVNR--RK-LSPEL---MNQLVP-E----------K--  296
OAT2_TM       LAVTLPCAPGILSLWWVPESARWLLTQGHVKEAHRYLLHCARLNG--RP-VCEDS---FSQEAV-SKVAAGE-R------ 309
OAT6_TM       FAISAPFLIFFLYSWWLPESSRWLLLHGKSQLAVQNLQKVAAMNG--RK-EEGER---LTKEVM-SSYIQSE-F--AS--  303
OAT3_TM       LTVSIPFFVFFLSSWWTPESIRWLVLSGKSSKALKILRRVAVFNG--KK-EEGER---LSLEEL-KLNLQKE-I--SL--  290
OAT1_TM       LLVSAPFFAFFIYSWFFIESARWHSSSGRLDLTLRALQRVARING--KR-EEGAK---LSMEVL-RASLQKE-L--TM--  302
OAT5_TM       VVASVPFFVFFLLSRWLVESARWLIITNKLDEGLKALRKVARTNG--IK-NAEET---LNIEVV-RSTMQEE-L--DA--  313
OAT7_TM       LVVSVPYFVIFLTSSWLLESARWLIINNKPEEGLKELRKAAHRSG--MK-NARDT---LTLEIL-KSTMKKE-L--EAA- 316
URAT1_TM      LVVSVPFFLCFLYSWWLAESARWLLTTGRLDWGLQELWRVAAING--KG-AVQDT---LTPEVL-LSAMREE-L--SM--  313
OAT4_TM       LAASVPFFAISLISWWLPESARWLIIKGKPDQALQELRKVARING--HK-EAK-N---LTIEVL-MSSVKEE-V--AS--  308
TM Domains    -TM6-----------]i
```

Figure S4 continued. Amino acid sequence alignment of the putative organic cation transporters and putative organic cation transporter novel from *Aedes aegypti* with human SLC22A in Bold. Similarity and identity threshold was set at 70%. The top line indicates the strongly conserved regions among SLC22A peptides "^" and positions of class specific amino acids "*". The bottom line indicated the predicted transmembrane domains by CCTOP where "i" signals intracellular loops and "o" signals extracellular loops.

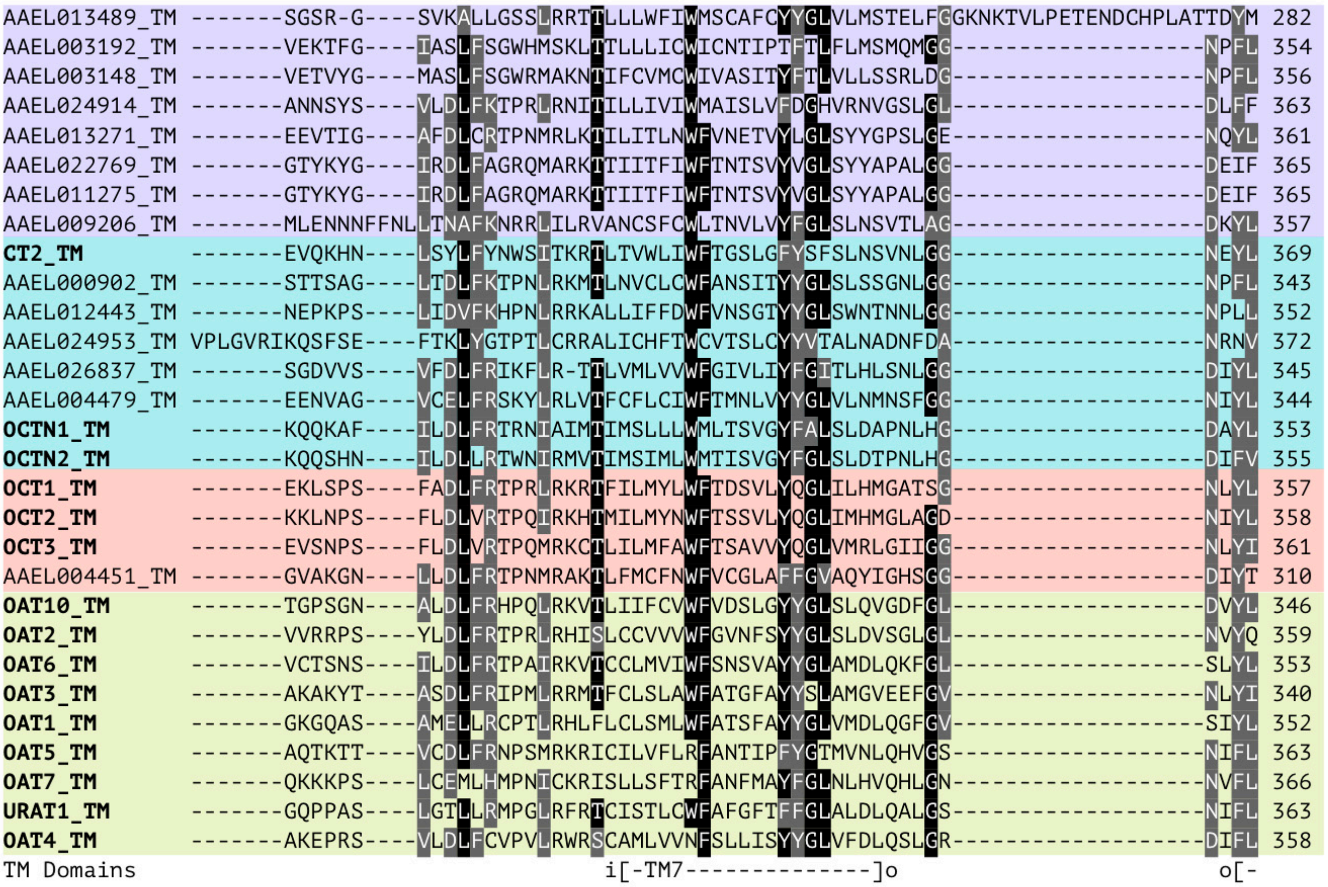

Figure S4 continued. Amino acid sequence alignment of the putative organic cation transporters and putative organic cation transporter novel from *Aedes aegypti* with human SLC22A in Bold. Similarity and identity threshold was set at 70%. The top line indicates the strongly conserved regions among SLC22A peptides "^" and positions of class specific amino acids "*". The bottom line indicated the predicted transmembrane domains by CCTOP where "i" signals intracellular loops and "o" signals extracellular loops.

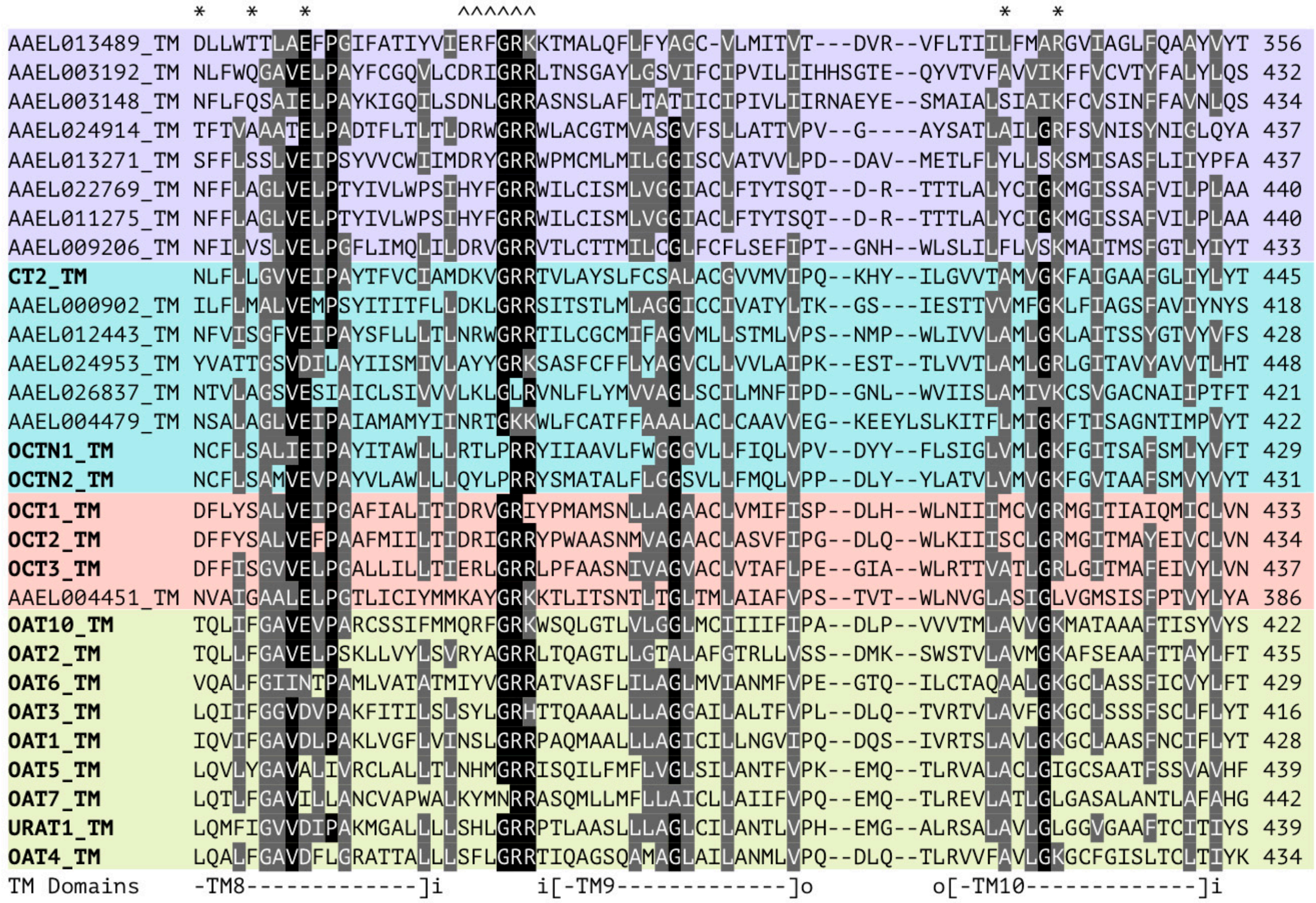

Figure S4 continued. Amino acid sequence alignment of the putative organic cation transporters and putative organic cation transporter novel from *Aedes aegypti* with human SLC22A in Bold. Similarity and identity threshold was set at 70%. The top line indicates the strongly conserved regions among SLC22A peptides "^" and positions of class specific amino acids "*". The bottom line indicated the predicted transmembrane domains by CCTOP where "i" signals intracellular loops and "o" signals extracellular loops.

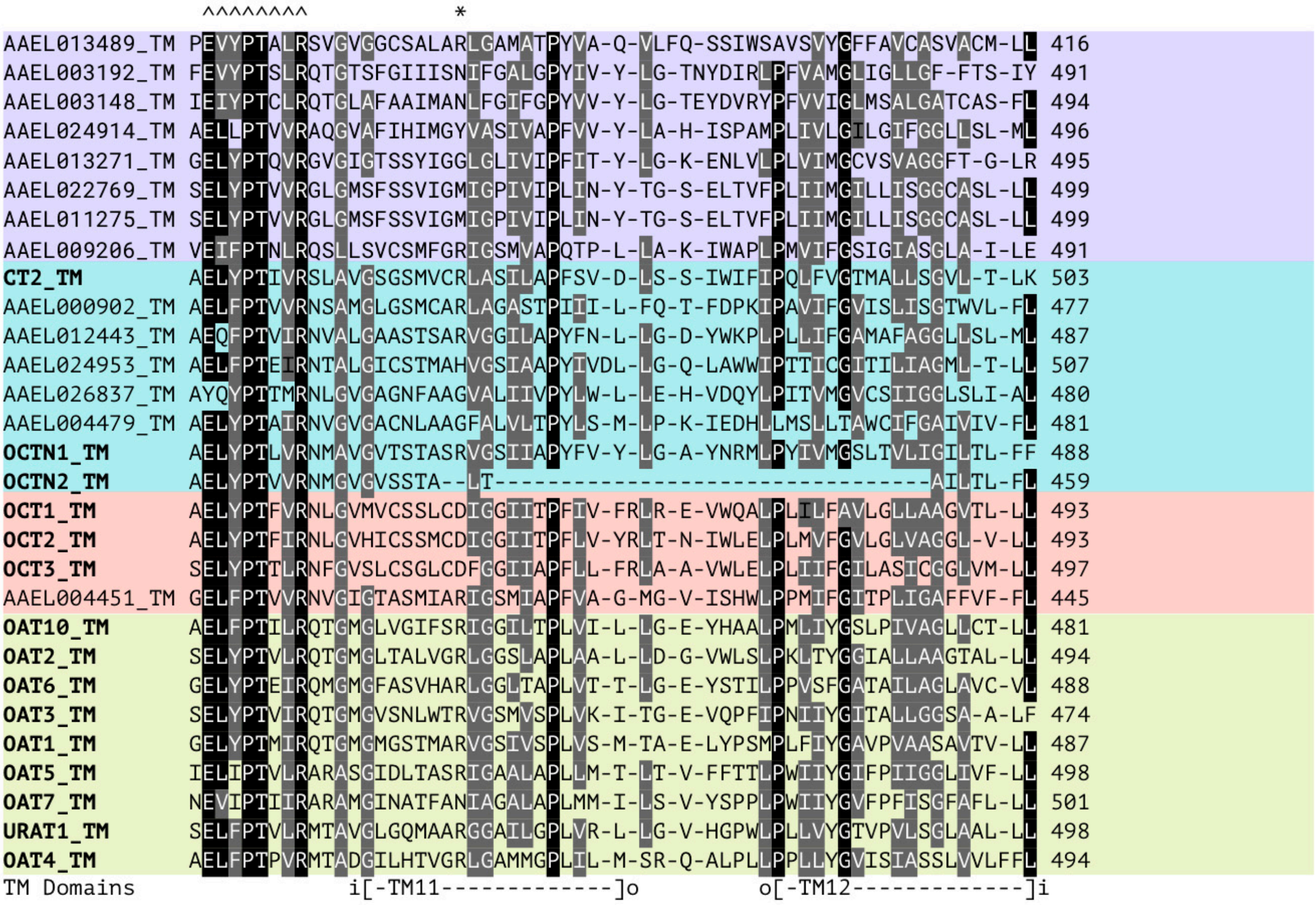

Figure S4 continued. Amino acid sequence alignment of the putative organic cation transporters and putative organic cation transporter novel from *Aedes aegypti* with human SLC22A in Bold. Similarity and identity threshold was set at 70%. The top line indicates the strongly conserved regions among SLC22A peptides "^" and positions of class specific amino acids "*". The bottom line indicated the predicted transmembrane domains by CCTOP where "i" signals intracellular loops and "o" signals extracellular loops.

Table S1. Primer sequences for qPCR. (Note "¦" indicates the exon-exon junction in amplicon.)

Gene	Sense Primer<br>Antisense Primer	Amplicon
AAEL004451	TGGTTAGTTTCGTTAGGCTGG<br>GTCGCTCTTTTTCGCAGTTG	TGGTTAGTTTCGTTAGGCTGGTTGTGGATACCGTTGAGAAGTGGGTGGCGAAAGATGGGCGAATCTA<br>ACATTGAAATGCGCGGTCGAACGGAAGACGGAG¦TGACCGCAACTGCGAAAAAGAGCGAC
AAEL012443	GTCTCCTGTCGTTGATGCTG<br>ATCTCTTCGGCAGTCTTGTTC	GTCTCCTGTCGTTGATGCTGCCGGAAACGCACAACCAGAAGCTACCGGAAACGATTGCCGATGGGGA<br>AAACTTTGGCAAGGTGAAAGTCATGCCCGGCGAAGAGGGTCGCCGGGGGGACGTGGAGAACAAGACT<br>GCCGAAGAGAT
AAEL000902	GCGGTTGATCATACACTGGAG<br>GAATTGGAATGCCTGGTAACG	GCGGTTGATCATACACTGGAGGAGCTGATGGGAATGTTGG¦<br>GCGACTTCGGTCGTTACCAGGCATTCCAATTC
AAEL024953	TGTTGCTTGGACGGATCG<br>AAGGCAATACTCATCCACGAC	TGTTGCTTGGACGGATCGGGCTGGGTGCTTCCGCTTCCGGAGTTTTCTACCCGGCGTTTGCGTTGT¦<br>TGACTGAAAACATCGGAACCCGTCATCGGTCGTGGATGAGTATTGCCTT
AAEL026837	TGGGTGATTATCTCGTTGGC<br>TTGGTAGGCTGTAAACGTCG	TGGGTGATTATCTCGTTGGCTATGATAG¦<br>TCAAGTGTTCCGTAGGGGCGTGCAATGCTATCATTCCGACGTTTACAGCCTACCAA
AAEL004479	AACTGTACCCAACTGCCATC<br>ATATACACCAAGCCGTGAGC	AACTGTACCCAACTGCCATCAGGAACGTTGGCGTGGGGGCTTGTAATCTCGCGGCTGGATTCGCTTT<br>AGTGCTTACCCCTTATCTTTCCATGTTG¦<br>CCAAAGATCGAGGATCATCTTCTTATGTCCCTGCTCACGGCTTGGTGTATAT
RPS17<br>*(AAEL004175)*	GATTTCCACACGAACAAGCG<br>TGCAGCTTGATGGAGATACC	GATTTCCACACGAACAAGCGGATCGTCGAAGAAGTGGCCATCATTCCAACGAAGCCCCTGCGCAACA<br>AGATCGCTGGG¦<br>TTTCGTGACACATCTGATGAAGCGCCTGCGCCACTCCCAGGTCCGTGGTATCTCCATCAAGCTGCA
ACT<br>*(AAEL011197)*	CGTTCGTGACATCAAGGAAA<br>GAACGATGGCTGGAAGAGAG	CGTTCGTGACATCAAGGAAAAACTGTGCTACGTTGCACTGGACTTTGAACAGGAAATGGCCACCGCT<br>GCCTCGTCCTCCTCCCTGGAGAAATCGTACGAACTTCCCGATGGACAAGTCATCACCATCGGCAATG<br>AACGTTTCCGTTGCCCAGAGGCTCTCTTCCAGCCATCGTTC

Table S2. Thermocycle utilize for qPCR.

**Step**	**1**	**2**	3	**4**	**5**	**Repeat 40X Steps 3-5**	**6**	**7**	**8**
Phase	Activation		Amplification				Melt Analysis		
Temp (°C)	50	50	95	95	95	95	95	95	95
Time (sec)	95	95	53	53	53	53	60	60	60
Trans. Rate (°C/s)	120	120	60	60	60	60	95	95	95
